# Using telecare in the development of learning disability services in Gloucestershire

**Published:** 2012-06-15

**Authors:** Christopher Haynes

**Affiliations:** Gloucestershire County Council, UK

**Keywords:** partnerships, value for money, independence, commissioning, success

## Abstract

Gloucestershire Learning Disability Partnership is a collaborative service of NHS Gloucestershire and Gloucestershire County Council. It serves over 2500 people with a learning disability and is currently working to incorporate telecare and telehealth into its mainstream services. We believe this proposed presentation would fit under the Developing Applications at Scale category. The Partnership Commissioners recognising the internal barriers to effecting telecare and telehealth applications commissioned a service from external providers which substantially improved service and reduced costs as compared with in-house telecare assessments and implementation. A new partnership was established with two external providers: Allied Health care/Tunstall. Their work is exemplified in the attached case study. The Learning Disability Partnership is now able to commission the evaluation of requirements, establishing the baseline and effecting the necessary changes in a continuous stream. This is now being rolled out across the county for learning disability services.

The presentation would follow the following format:

- The barriers to success: the internal barriers which prevented telecare and telehealth from happening. How we get in our own way to making change happen.
- How we commissioned around the barriers and achieved success.
- The case study and other examples of significant service improvements and savings.
- The next generation:
  At the front end of services the partnership is now actively using teleprompting, geofencing and GPS systems to facilitate the enablement of people with a learning disability and enhance their abilities to be independent. Service users express delight in having control of their own lives versus having ‘support workers’ follow them around (DVD available).
- Q and A. The main presenter would be Chris Haynes, an accomplished international speaker winner of the Queens Golden Jubilee Medal for Public Service and currently Joint Commissioner of Learning Disability Services.

There will be representatives from Allied and Tunstall on hand to discuss their involvement and how it works.

